# Simple Liver Cyst as a Focus of *Salmonella paratyphi* Abscess: A Case Report

**DOI:** 10.1155/2009/456810

**Published:** 2009-12-06

**Authors:** A. Sangwaiya, A. Patel, J. Chan, J. Arnold

**Affiliations:** Department of Gastroenterology, Ealing Hospital NHS Trust, London UB1 3HW, UK

## Abstract

Salmonellosis, endemic in various part of the world, is considered a differential diagnosis in a tropical traveller. Although it usually presents as gastroenteritis, its various clinical syndromes may vary from mild gastroenteritis to severe septicaemia including abscess formation, the later two being the most common cause of morbidity and mortality. Here we present a patient who returned to the UK after an overseas trip and was diagnosed with a pyogenic liver abscess with *Salmonella paratyphi* at a site of a pre-existing simple liver cyst.

## 1. Introduction

Salmonellosis is a well-known cause of morbidity and mortality in patients, particularly in endemic areas. It is also a well-known cause of pyogenic liver abscess [1, 2]. It is frequently encountered in patients with pre-existing liver pathology such as amoebic abscess or echinococcus cyst [3].

We present a case of salmonellosis complicated by pyogenic liver abscess in a previously known simple liver cyst. Such a case has been reported only once in the English literature [4]. This signifies the importance of the fact that simple cysts can become a focus of abscess formation.

## 2. Case Presentation

A 74-year-old gentleman presented to the Accident and Emergency Department following a 3-month stay in India. Four weeks into his return, he started developing daily episodes of fever and night sweats. Whilst in India, he was asymptomatic although he did not take any malaria prophylaxis or salmonella vaccine during his travel. 

The patient had no comorbidities except for a renal calculi detected in 2004. He was also known to have a simple cyst measuring 5 cm in diameter in the right lobe of the liver, incidentally found on routine abdominal ultrasonography (USG). He did not take any regular medication, and was otherwise in good health.

On examination, he had a temperature of 38.2°C, pulse 90 beats per minute regular, and blood pressure of 116/60 mmHg. Systemic examinations of cardiovascular, respiratory, abdominal, and neurological systems were unremarkable.

His initial blood biochemical examination showed normal liver and kidney functions but a raised c-reactive protein (CRP) of 129 mg/dL. His haematological parameters showed a raised white cell count of 12 000/cumm with predominant neutrophilia. No malarial parasites were detected. Chest X-ray did not show any signs of underlying infection. 

Repeated blood and urine cultures were obtained and abdominal USG requested to find any abdominal collection as a source of sepsis. Our differential diagnosis at this stage was malaria, salmonellosis, or amoebiasis. The delayed start of his symptoms vis a vis his travel history made viral aetiology, namely, dengue fever unlikely. 

His blood culture grew *Salmonella paratyphi A. *Serologies for amoeba and hydatid disease were negative. Abdominal USG showed a collection in the right liver lobe, the same site as the simple cyst noted on the previous scans. The area was significantly larger than previously noted with an underlying abscess. Abdominal and pelvic CT scan did not reveal any further abscesses.

The liver abscess was drained under ultrasound guidance using a pigtail catheter and the aspirate cultures also grew *Salmonella paratyphi A*. The patient received systemic antibiotic, Ceftriaxone 2 gm intravenously once daily for 10 days based on the sensitivity report and made an uneventful recovery. On followup, he remains asymptomatic.

We present a case of liver abscess caused by *Salmonella paratyphi A*, in a previously known simple cyst of the liver. Such a case has been reported only once previously. 

## 3. Discussion

Salmonellosis is endemic in the developing world and a major public health problem. It usually presents as gastroenteritis, but may manifest as various clinical syndromes [5]. The disease severity varies from mild self-limited gastroenteritis to septicaemia and abscess formation, the later being a major cause of morbidity and mortality [5]. 

Salmonella infection can involve any part of the abdomen, but most commonly involves the hepatobiliary system and spleen [6]. Certain conditions such as malignancies, sickle cell disease, liver cirrhosis, and gastric achlorhydria predispose to intra-abdominal salmonella sepsis [7–9]. 

Salmonellosis is often diagnosed on blood cultures and stool cultures. Pyogenic liver abscess (PLA) is a rare presentation and most frequently caused by *Bacteroides, Enterococcus, E. coli, Klebsiella, Streptococcus, Staphylococcus, and Salmonella spp *[10]. It is diagnosed by ultrasound guided sampling of collection followed by culture and repeated blood cultures [11]. It is occasionally difficult to diagnose pyogenic liver disease as in up to 50% of the PLA, blood cultures are negative. It is important to consider an alternative diagnosis of amoebic liver disease in a traveller returning from tropics as was our patient. His serology for *Entamoeba histolytica* was negative. Absence of antibodies after one week of symptoms is almost exclusive of amoebic liver disease and stool examination for detection of cysts is recommended in case patient is a carrier for the infection. Liver abscess has also been described over liver metastases and hepatocellular carcinoma [12]. Tumours of the hepatobiliary region can occasionally mimic liver abscess whilst an underlying malignancy on liver abscess is associated with poor prognosis [12–14]. There has also been a case report of salmonella in polycystic liver disease. In one case report of polycystic liver disease, one of the cysts became infected with *Salmonella javiana* that was successfully treated with percutaneous drainage and systemic antibiotic followed by installation of intracystic tetracycline as a sclerosant thus preventing a laparotomy [15]. Similarly PLA caused by *Salmonella enteritidis* has been reported in a returned traveller with HIV infection [16].

Many strategies have been proposed for treatment of pyogenic liver abscesses depending on their initial size on CT or ultrasound abdomen. Some studies suggest that small abscesses (less than 3 cm) should be treated with intravenous antibiotics alone [17]. Large uniloculated (more than 3 cm) abscesses should be treated with intravenous antibiotics and percutaneous drainage in the form of ultrasound guided needle aspiration or percutaneous catheter drainage. Surgical or laparoscopic drainage has been recommended for large (more than 3 cm) multiloculated collections. Other studies suggest treatment with antibiotics alone on abscesses less than 5 cm, and percutaneous drainage or surgery for sizes more than or equal to 5 cm [18]. 

Our case illustrates the value of percutaneous ultrasound guided drainage either by needle aspiration or by catheter drainage to reduce the time for resolution of sepsis and prevention of mortality. It also illustrates the fact that simple liver cysts can be a focus of pyogenic liver abscess and can be treated effectively with percutaneous catheter insertion and intravenous antibiotic therapy directed by microbiological sensitivities.
